# Altered expression of KLC3 may affect semen parameters

**Published:** 2016-01

**Authors:** Pegah Kargar- Dastjerdy, Marziyeh Tavalaee, Mansoor Salehi, Mojtaba Falahati, Tayebeh Izadi, Mohammad Hossein Nasr Esfahani

**Affiliations:** 1 *Department of Reproductive Biotechnology, Reproductive Biomedicine Research Center, Royan Institute for Biotechnology, ACECR, Isfahan, Iran.*; 2 *Cell and Molecular Department, Pharmaceutical Sciences Branch, Islamic Azad University, Tehran, Iran.*; 3 *Department of Genetics and Molecular Biology, Medical School, Isfahan University of Medical Sciences, Isfahan, Iran.*; 4 *Department of Nanotechnology, Pharmaceutical Sciences Branch, Islamic Azad University, Tehran, Iran.*; 5 *Department of Cellular Biotechnology, Cell Science Research Center, Royan Institute for Biotechnology, ACECR, Isfahan, Iran.*; 6 *Isfahan Fertility and Infertility Center, Isfahan, Iran.*

**Keywords:** *KLC3*, *Infertility*, *Sperm parameters*, *Oligozoospermia*, *Asthenozoospermi*

## Abstract

**Background:**

KLC3 protein as a member of the kinesin light-chain protein family plays an important role in spermatogenesis, during formation of mitochondrial sheath in the mid piece of the sperm tail.

**Objective:**

This study for the first time aims to compare the expression of the KLC3 gene between fertile and infertile individuals.

**Materials and Methods:**

Semen samples were collected from 19 fertile individuals who were selected from embryo-donor volunteers and 57 infertile individuals who had abnormal sperm parameters according to world health organization criteria. Sperm parameters using computer assisted sperm analysis and the quantitative KLC3-gene expression using the real-time PCR method were measured.

**Results:**

Our results revealed a significant correlations between sperm concentration with relative expression of KLC3 only in infertile groups (r=0.45, p=0.00). A significant correlation was not found between KLC3 expression and sperm motility; however, the relative expression of KLC3 was significantly higher in asthenozoospermic compared to non-asthenozoospermic individuals.

**Conclusion:**

Low expression of KLC3 may result in improper function of midpiece, which has important function in sperm motility. The results of this study show that aberrant expression of KLC3 might be associated with phenomena like oligozoospermia and asthenozoospermia. This article is extracted from student’s thesis.

## Introduction

Round spermatids differentiate to fully mature spermatozoon, composed of head, midpiece and tail (1, 2). The mammalian sperm midpiece is characterized by mitochondrial helical sheaths surrounding the axonemal complex and nine outer dense fibers (ODFs) (3). Several proteins are associated with these structures, including sperm-associated antigens (Spags), Spetex- 1, Tektins and kinesins (4-6).

Kinesins are heterotetrameric motor molecules, comprised of two heavy chains (KHCs) and two light chains (KLCs). They act as a mechano-chemical enzyme involved in the intracellular transport of organelles such as mitochondria to the midpiece, during spermiogenesis (7-9). Originally, coding strand of KLCs was cloned from rat brain (9). Later on, analysis of KLC family revealed that it has a conserved middle and divergence N- and C-termini regions. The highly conserved region consists of heptad repeats (HRs) and tandem tetra-tricopeptide repeats (TPRs). The HRs facilitates binding of heavy and light chains (10, 11).

**Figure 1:**
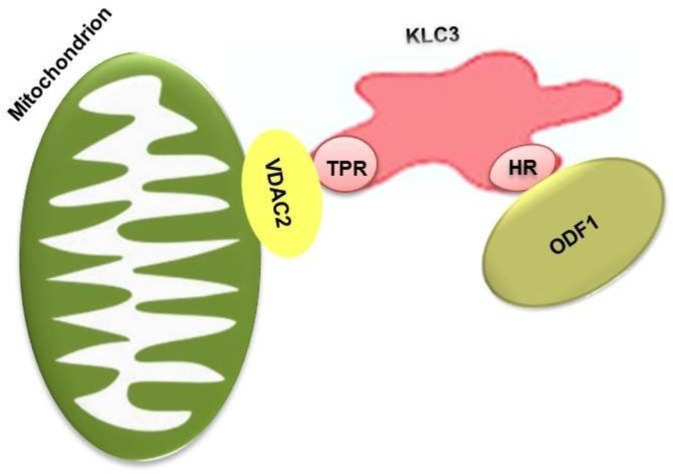
Schematic presentation of KCL3 role which acts as an anchor protein for attachment of mitochondria to ODF: the HR region of KLC3 interacts with the leucine zipper domain of ODF1 protein. The TPR region associates with mitochondrial outer membrane protein: VDAC2. ODF: Outer Dense Fibers, HR: Heptad Repeat, VDAC2: Voltage-Dependent Anion Channel 2

KLC3 is a testis specific protein and expressed in post-meiotic male germ cells (12). Upon expression of kinesins, mitochondria are transported to midpiece, to bind to ODF via KLC3. Therefore, KLC3 acts as an anchor protein for bringing mitochondria to ODF (13). Binding of mitochondrial membrane to KLCs is through mitochondrial protein known as Voltage-Dependent Anion Channel 2 (VDAC2) (Figure 1). These events result in accumulation of mitochondria, which are tightly bound to ODF in sperm tail midpiece and eventually lead to formation of mitochondrial sheaths, which are essential for sperm motility and thereby fertility (14, 15). 

Initial clustering of mitochondria occurs in a microtubule-dependent fashion via conventional kinesins and concomitant with mitochondrial aggregation. During this process, the conventional kinesins are replaced by kinesins containing KLC3 and it finally leads to tight attachment of mitochondria to ODF (16). In this regard, the mitochondria of heptad repeat motifs knockout mice (KLC3ΔHR), despite having the clustering of mitochondria, does not bind to ODF and this event results in reduction of sperm count, motility, and abnormal midpiece (14). Sperm, despite being transcriptionally inactive, contains numerous RNAs. Some of these RNA may play an active role during early embryogenesis while others are remnant of RNA, which have played different role during spermiogenesis. Researchers have shown that some of these transcripts may act as biomarkers for functional assessment of a semen quality (17). 

Therefore, based on this background, it was initially assessed expression of KLC3 in fertile individuals, to obtain the normal range of expression of KLC3. Then, it was categorized infertile individuals based on the normal range of expression of KLC3. Subsequently, the semen parameters were compared between individuals with normal and abnormal expression of KLC3.

## Materials and methods

**Figure 2:**
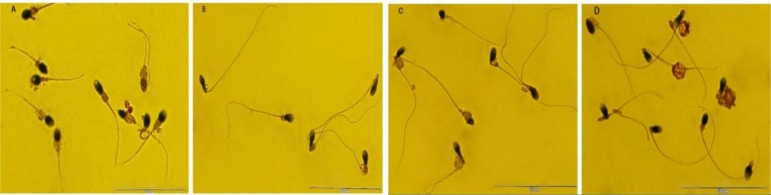
Three asthenoteratozoospermic (A, B, C) and one asthenozoospermic (D) individuals were deliberately selected based on WHO-2010 criteria with high percentage of sperm with abnormal midpiece. Scale bars, 50 µm (A-D).

This case-control study was approved by the Institutional Review Board of Royan Institute and Isfahan Fertility and Infertility Center (IFIC), Isfahan, Iran from 2014-2015. Fifty seven semen samples of infertile individuals referring to andrology unit of IFIC were randomly selected and included in this study. One of these individuals was asthenozoospermic and three asthenoteratozoospermic individuals were deliberately selected based on World Health Organization (WHO) criteria (18) with high percentage of sperm with abnormal midpiece (Table I, Figure 2). Fertile individuals (n= 19) were recruited from couples participating in the embryo donation program. Informed consent was obtained from all of the individuals participated in this study.

**Semen analysis**

Samples were collected by masturbation into a sterile specimen container following a 3-5 days of sexual abstinence. Semen samples were allowed to be liquefied for a period of 15–30 minutes at room temperature and using computer- aided sperm analysis (CASA) were analyzed for sperm concentration, motility, and morphology based on the guidelines of the WHO (2010) (18). Semen analysis was carried out by one trained individual. 

**RNA extraction and first strand synthesis**

Total RNA from semen samples of both fertile and infertile individuals were extracted using TRIzol. The integrity of extracted RNA was evaluated by agarose gel electrophoresis and its concentration was calculated by measuring absorbance at 260 nm. In order to eliminate possible contamination of genomic DNA, RNA- containing samples were treated with DNaseI (Fermentas). First strand cDNA synthesis was carried out using 2 μg of total RNA with the RevertAid First Strand cDNA Synthesis kit (Fermentas) according to the manufacturer’s protocol.

**Real-time polymerase chain reaction (qPCR)**

Real- time PCR was carried out according to the manufacturer’s protocol (TaKaRa) in a thermal cycler step one plus applied Biosystems (ABi). The PCR mixture for each reaction contained 10μl SYBR premix Ex Taq II (TaKaRa), 1μl of each primer (5 pmol/μl) and 50 ng cDNA adjusted to a final volume of 20 μl using dH2O. All reactions were carried out in Triplicate. Real- time- specific primer pairs were designed by the Beacon designer 7.5. The real- time PCR protocol was composed of: 30 sec at 95^o^C followed by 40 repetitive cycles for 5 sec at 95^o^C, 10 sec at 60^o^C and 30 sec at 72^o^C. The expression level of KLC3 mRNA was normalized by expression of the housekeeping gene GAPDH. The calculation of relative expression was assessed using the ΔΔCt method (19, 20).

**Statistical analysis**

Microsoft Excel and Statistical Package for the Social Sciences, SPSS Inc., Chicago, Illinois, USA (SPSS) were used to data analyze. Data are expressed as means±SEM (standard error of mean). Difference in gene expression between groups was assessed using one-way ANOVA. Two-tailed Pearson correlation test was used to assess the correlations between parameters. P<0.05 was considered as significant. 

## Results

**Figure 3:**
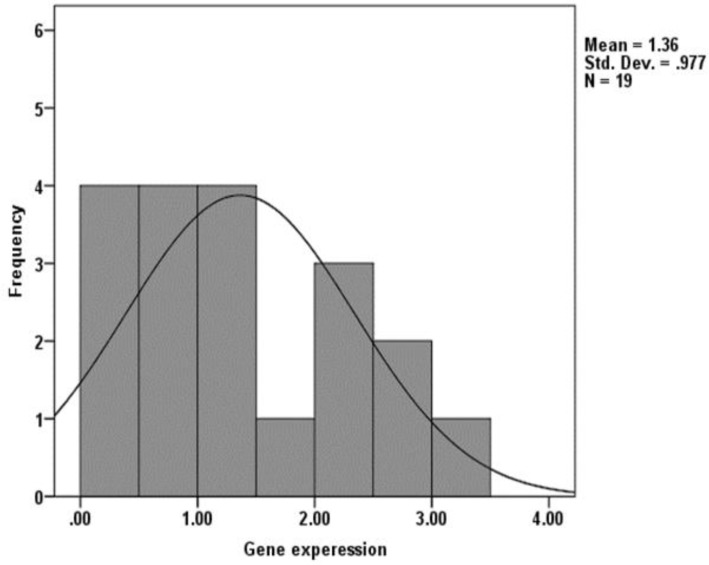
Assessment of the distribution of *KLC3* expression in fertile individuals using the lower and upper 10 percentiles.

Following the assessment of relative KLC3 expression in 19 fertile and 57 infertile individuals, the distribution of KLC3 in fertile individuals were defined lower and upper 10 percentiles. This range of KCL3 expression was taken as normal range of expression for KLC3, which was between 0.2-2.9 (Figure 3). The bases of defining 10 percentile were according to the method used for defining lower limits of sperm parameters by WHO (18). 

Based on this range, the individuals were categorized into 3 subgroups (Table II): individuals with relative expression of KLC3 lower than 0.2 (n= 4), within the defined range of 0.2-2.9 (n= 36) and higher than 2.9 (n= 17). Semen parameters were compared between groups II and III. Comparison between group I with II and III was not carried out due to low sample number. 

**Figure 4:**
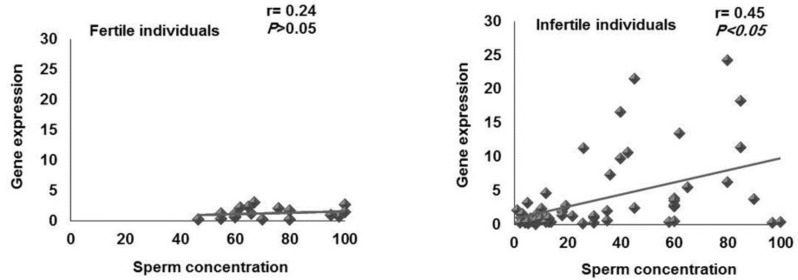
Correlation between sperm concentration and relative *KLC3* expression in the fertile (n= 19) and infertile (n= 57) individuals

The mean of sperm concentration in the group I, II and III were 13.06±6.41, 24.2±4.24 and 53.74±6.27, respectively. Comparison of sperm concentration was carried out between groups II and III, which showed individuals with relative expression of KLC3 within the defined ranges (group II) had significantly (p=0.00) lower mean sperm concentration than individuals with relative expression of KLC3 higher than 2.9. Based on this result, the correlation between sperm concentration with relative KLC3 expression was carried out in both fertile (r= 0.24, p= 0.33) and infertile groups (r= 0.45, p= 0.00), which revealed a positive correlation between these two parameters in both fertile and infertile individuals, that was significant only for infertile group (Figure 4).

The mean of sperm motility in the groups I, II and III were 15.22±5.45, 30.14±3.58 and 27.95±4.27, respectively. No significant difference was observed between groups II and III. Correlation analysis between sperm motility with relative KLC3 expression revealed no significant correlation between these two parameters in both fertile (r= 0.09, p= 0.70) and infertile (r= -0.13, p= 0.34) individuals. The mean of abnormal sperm morphology in the groups I, II and III were 97.5±1.19, 97.47±0.25 and 96.82±0.51, respectively and no significant difference was observed between groups II and III. Correlation analysis between abnormal sperm morphology with relative KLC3 expression showed no significant correlation between these two parameters in both fertile (r= 0.1, p=0.67) and infertile (r= -0.05, p= 0.69) individuals. 

Since KLC3 is involved in formation of mitochondrial sheaths associated with sperm midpiece, the mean percentage of abnormal sperm midpiece in groups I, II and III were evaluated. The mean values for percentage abnormal sperm midpiece were 20.00±5.29, 23.12±2.3 and 27.05±4.71, respectively. Comparison was only carried out between groups II and III and there was not a statistically significant difference between them.

**Figure 5:**
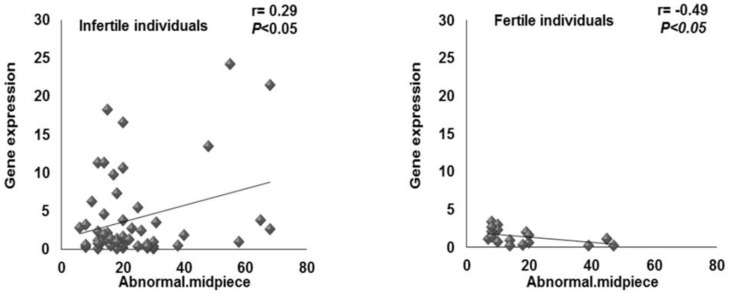
Correlation between percentage of abnormal sperm midpiece and relative *KLC3* expression in the fertile (n= 9) and infertile (n= 57) individuals

Correlation analysis between percentage of abnormal sperm midpiece with relative KLC3 expression was carried out in both the fertile (r= -0.49, p= 0.039) and infertile (r= 0.29, p= 0.036) individuals, which revealed a significant positive correlation only between these two parameters only in infertile individuals (Figure 5).

**Figure 6:**
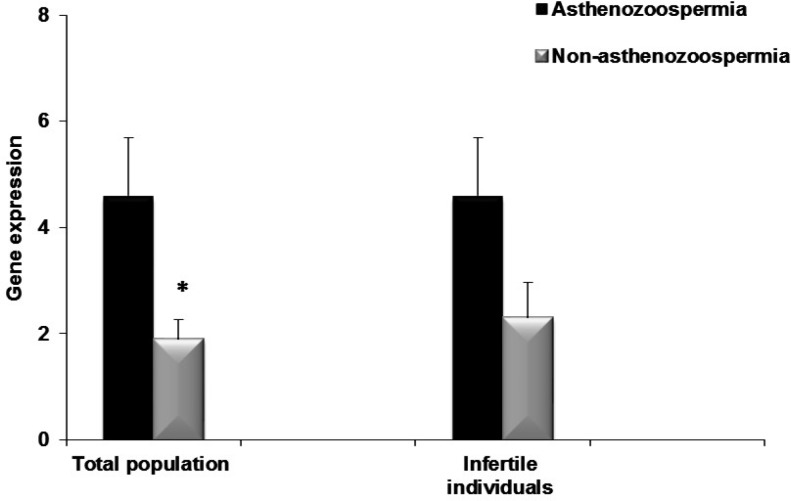
Comparison of relative expression of *KLC3* between asthenozoospermic and non- asthenozoospermic individuals

Considering the role of KLC3 in formation of mitochondrial sheaths, which has important function in motility, infertile individuals were categorized into asthenozoospermic and non-asthenozoospermic individuals. Based on total motility less than 40% according to WHO-2010 as well as compared relative expression of KLC3 (Figure 6) between two groups. A significant difference was observed in the total population (4.6±1.09 vs. 1.9±0.37, p= 0.023) and it was almost significant in the infertile individuals (4.6±1.09 vs. 2.3±0.67, p= 0.09). 

**Table I:** Description of sperm parameters and expression of *KLC3* in four infertile individuals deliberately included

**Case**	**Concentration (10** ^6^ **/ml)**	**Abnormal morphology%**		**Motility%**	**Relative ** ***KLC3*** ** expression**
		**Head**	**Neck**		
1	50	98	78	15	21.49
2	40	100	68	30	2.61
3	80	99	55	15	24.18
4	62	96	48	5	13.42

**Table II:** Comparison of semen parameters between infertile individuals with normal and abnormal range of relative KLC3 expression (N=57)

**Semen parameters**	**Relative ** ***KLC3*** ** expression**			
	**0-0.2 (N= 4)**	**0.2-2.9 In range (N= 36)**	**> 2.9 (N= 17)**	**p-Value** *
Sperm concentration (10^6^/ml)	13.06±6.41	24.2±4.24	53.74±6.27	0.000
Sperm motility (%)	15.22±5.45	30.14±3.58	27.95±4.27	0.71
Abnormal sperm morphology (%)	97.5±1.19	97.47±0.25	96.82±0.51	0.21
Abnormal midpiece (%)	20±5.29	23.12±2.3	27.05±4.71	0.40

* Comparison was only carried out between groups II and III.

## Discussion

Recent advance in andrology has focused on transcriptome present of the sperm. Two main roles have envisaged for these transcripts, considering inadequate translation machinery present in sperm: 1) the transcripts are remnants of RNA produced during spermiogenesis before sperm chromatin condensation stage and 2) some of these RNAs may play critical role during early embryogenesis (17, 21-23). The example of latter is Clusterin, involved in cell-cell and cell-substratum interactions, lipid transportation, membrane recycling, stabilization of stress proteins, and promotion or inhibition of apoptosis, while the example of former is RNA for protamine 2 (17, 24). The levels of expression of these RNAs have been shown to be associated with their protein levels and functions. Therefore, deficiency or lack of transcripts could be associated with sperm functional anomalies and infertility (25-27). Considering KLC3 involve in anchoring the mitochondria to ODF1, and through this approach, stabilizes the structural integrity of organelles in the midpiece, we aimed to compare the expression of the KLC3 gene between fertile men and infertile individuals. Sperm derived from knockout mice model showed reduced sperm count, motility and abnormalities in midpiece region (14). Despite evident role of KLC3 in mice, to our knowledge, there was no study, which has assessed the role of KLC3 in human. Therefore, the relative expression of KLC3 was evaluated in fertile and infertile individuals. 

The results of this study revealed that relative expression of KLC3 ranges between 0.2-3.42 in fertile individuals (Figure 3), therefore based on previous studies, the range of expression was taken between 10 to 90 percentiles as the normal range of KLC3 expression, which was 0.2-2.9. So, the infertile individuals were classified into three groups based on this criterion; individuals with normal level of KLC3 expression, higher or lower than normal range of expression (28, 29). 

Out of 57 infertile individuals, 4 and 17 showed lower and higher level of KLC3 expression, respectively, and 36 individuals were within the normal range. Based on this classification, the semen parameters in individuals with high and normal level of KLC3 expression were assessed and compared (The comparison between low and normal group was not assessed, due to low number of individuals).

Among all semen parameters, only the sperm concentration was significantly higher in individuals showing high expression of KLC3 compared to individuals whose KLC3 expression was within normal range. Furthermore, only a significant correlation was observed between sperm concentration and relative expression of KLC3 and not with the other semen parameter (Figure 4). These data may suggest that expression of KLC3 might be considered as a rate limiting factor in the process of spermatogenesis. To ensure that the observed result is not an artifact of RNA quality between two groups, the cycle of threshold for the house keeping gene between two groups were compared and the results revealed similar RNA quality between these two groups.

It is of note that previous studies have shown a significant correlation between testis specific transcripts, like phospholipase zeta, protamine 1, CAPZA3, and Fank1 with sperm concentration (30, 31). Therefore, it is likely that low expression of testis transcript is associated with efficiency of spermatogenesis. However, there is a contrary report for other transcripts (32). 

Hence, such conclusion needs further validation. It was interesting that it was observed a positive significant correlation between abnormal sperm midpiece and the relative expression of KLC3 in infertile individuals (Figure 5). It is like that in these individuals, the expression of KLC3 might have been defective and there might be a negative feedback for production of higher amount of KLC3.

An alternative explanation would be that higher presence of cytoplasmic remnants may account higher KLC3. However, further investigation at protein level is required to discriminate between these two possibilities. To add to these possibilities, the relative expression of KLC3 was substantially higher in the 4 infertile individuals, which were deliberately selected based on high percentage of midpiece defects (see Table I). In this study, although a significant correlation did not observe between KLC3 expression and sperm motility, the relative expression of KLC3 was significantly higher in asthenozoospermic compared with nonasthenozoospermic individuals (Figure 6). Low expression of KLC3 may result in improper function of midpiece, which has important function in sperm motility. 

## Conclusion

In conclusion, the results of this study state that aberrant expression of KLC3 might be associated with phenomena like oligozoospermia and asthenozoospermia.
